# 4-[(1,3-Thia­zol-2-yl)sulfamo­yl]phenyl 2,2,2-trifluoro­acetate

**DOI:** 10.1107/S1600536812011683

**Published:** 2012-03-24

**Authors:** Abdullah M. Asiri, Hassan M. Faidallah, Khalid A. Alamry, Seik Weng Ng, Edward R. T. Tiekink

**Affiliations:** a Chemistry Department, Faculty of Science, King Abdulaziz University, PO Box 80203, Jeddah, Saudi Arabia; b The Center of Excellence for Advanced Materials Research, King Abdulaziz University, Jeddah, PO Box 80203, Saudi Arabia; c Department of Chemistry, University of Malaya, 50603 Kuala Lumpur, Malaysia

## Abstract

In the title compound, C_11_H_7_F_3_N_2_O_4_S_2_, the 1,3-thia­zol-2-amine residue is almost perpendicular to the central benzene ring [dihedral angle = 84.3 (2)°]. There is a small twist between the benzene ring and the ester group [C—O—C—C torsion angle = 9.8 (6)°]. Thus, the mol­ecule has an L-shape. Inversion-related dimers are connected in the crystal packing by pairs of N—H⋯N hydrogen bonds formed between the amine H and thia­zole N atom *via* eight-membered {⋯HNCN}_2_ synthons.

## Related literature

For the biological efficacy of F and CF_3_ in medicinal chemistry, see: Fokin & Kolomiyets (1988 ▶); Bonacorso *et al.* (2006 ▶). For background to the biological applications of sulfonamides, see: Croitoru *et al.* (2004 ▶); Dogruer *et al.* (2010 ▶). For related structures, see: Asiri *et al.* (2011 ▶, 2012 ▶).
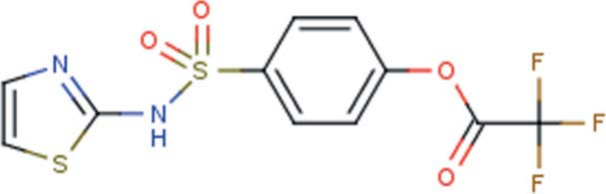


## Experimental

### 

#### Crystal data

C_11_H_7_F_3_N_2_O_4_S_2_
*M* *_r_* = 352.31
Monoclinic, 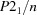

*a* = 8.7498 (5) Å
*b* = 14.4343 (9) Å
*c* = 10.7225 (5) Å
β = 96.749 (5)°
*V* = 1344.84 (13) Å^3^
*Z* = 4
Mo *K*α radiation
μ = 0.45 mm^−1^
*T* = 100 K
0.30 × 0.30 × 0.10 mm

#### Data collection

Agilent SuperNova Dual diffractometer with an Atlas detector
Absorption correction: multi-scan (*CrysAlis PRO*; Agilent, 2011 ▶) *T*_min_ = 0.876, *T*_max_ = 0.956
12068 measured reflections
3105 independent reflections
2252 reflections with *I* > 2σ(*I*)
*R*_int_ = 0.036

#### Refinement

*R*[*F* ^2^ > 2σ(*F* ^2^)] = 0.071
*wR*(*F* ^2^) = 0.215
*S* = 1.06
3105 reflections
199 parameters
H-atom parameters constrained
Δρ_max_ = 0.69 e Å^−3^
Δρ_min_ = −0.41 e Å^−3^

### 

Data collection: *CrysAlis PRO* (Agilent, 2011 ▶); cell refinement: *CrysAlis PRO*; data reduction: *CrysAlis PRO*; program(s) used to solve structure: *SHELXS97* (Sheldrick, 2008 ▶); program(s) used to refine structure: *SHELXL97* (Sheldrick, 2008 ▶); molecular graphics: *X-SEED* (Barbour, 2001 ▶) and *DIAMOND* (Brandenburg, 2006 ▶); software used to prepare material for publication: *publCIF* (Westrip, 2010 ▶).

## Supplementary Material

Crystal structure: contains datablock(s) global, I. DOI: 10.1107/S1600536812011683/hg5192sup1.cif

Structure factors: contains datablock(s) I. DOI: 10.1107/S1600536812011683/hg5192Isup2.hkl

Supplementary material file. DOI: 10.1107/S1600536812011683/hg5192Isup3.cml

Additional supplementary materials:  crystallographic information; 3D view; checkCIF report

## Figures and Tables

**Table 1:** Hydrogen-bond geometry (Å, °)

*D*—H⋯*A*	*D*—H	H⋯*A*	*D*⋯*A*	*D*—H⋯*A*
N2—H2⋯N1^i^	0.88	1.99	2.858 (5)	171

Symmetry code: (i) 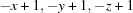.

**Fig. 1.**
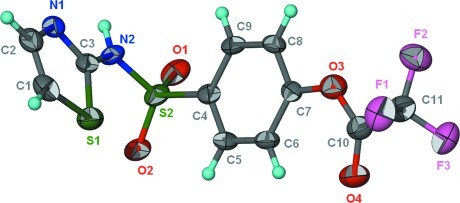
The molecular structure of (I) showing the atom-labelling scheme and displacement ellipsoids at the 50% probability level.

**Fig. 2.**
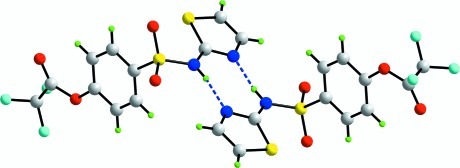
Centrosymmetric dimers in (I) sustained by N—H···N hydrogen bonds shown as blue dashed lines leading to eight-membered {···HNCN}2 synthons.

## supplementary crystallographic information

### Comment

The presence of fluoride and trifluoromethyl groups, in particular, has long been recognized in medicinal chemistry as a substituent of distinctive qualities (Fokin & Kolomiyets, 1988; Bonacorso *et al.*, 2006) owing to their ability to alter the physico-chemical and biological characteristics of molecules. In connection with on-going studies of sulphonamides, biological (Croitoru *et al.*, 2004; Dogruer *et al.*, 2010) and crystallographic (Asiri *et al.*, 2011; Asiri *et al.*, 2012), the title CF_3_-derivatized sulphonamide (I), was investigated.

In (I), Fig. 1, with reference to the central benzene ring, the 1,3-thiazol-2-amine residue occupies an almost perpendicular position with the N2—S2—C4—C5 torsion angle being 122.7 (3)°. The dihedral angle between the benzene and thiazol rings [r.m.s. deviation = 0.011 Å] is 84.3 (2)°. There is a small twist between the benzene ring and the ester group with the C10—O3—C7—C6 torsion angle being 9.8 (6)°. To a first approximation, the molecule of (I) has the shape of the letter *L*.

In the crystal packing, N—H···N hydrogen bonds are formed between the amine-H and thiazol-N atoms of centrosymmetrically related molecules to form eight-membered {···HNCN}_2_ synthons, Fig. 2 and Table 1. Molecules pack with no specific intermolecular interactions between them.

### Experimental

A mixture of sulfamerazine (2.6 g, 10 mmol) in THF (30 ml) and trifluroacetic anhydride (2.2 g, 11 mmol) was refluxed for 2 h. The solid which separated on cooling was recrystallized from ethanol. Yield: 68%. *M*.pt: 513–514 K.

### Refinement

Carbon-bound H-atoms were placed in calculated positions [N—H = 0.88 Å and C—H = 0.95 Å; *U*_iso_(H) = 1.2*U*_eq_(N,*C*)] and were included in the refinement in the riding model approximation. Owing to poor agreement, the (0 2 1) reflection was omitted from the final cycles of refinement.

### Crystal data

**Table:**

C_11_H_7_F_3_N_2_O_4_S_2_	*F*(000) = 712
*M**_r_* = 352.31	*D*_x_ = 1.740 Mg m^−^^3^
Monoclinic, *P*2_1_/*n*	Mo *K*α radiation, λ = 0.71073 Å
Hall symbol: -P 2yn	Cell parameters from 3265 reflections
*a* = 8.7498 (5) Å	θ = 2.3–27.5°
*b* = 14.4343 (9) Å	µ = 0.45 mm^−^^1^
*c* = 10.7225 (5) Å	*T* = 100 K
β = 96.749 (5)°	Irregular, light-yellow
*V* = 1344.84 (13) Å^3^	0.30 × 0.30 × 0.10 mm
*Z* = 4	

### Data collection

**Table:**

Agilent SuperNova Dual diffractometer with an Atlas detector	3105 independent reflections
Radiation source: SuperNova (Mo) X-ray Source	2252 reflections with *I* > 2σ(*I*)
Mirror monochromator	*R*_int_ = 0.036
Detector resolution: 10.4041 pixels mm^-1^	θ_max_ = 27.6°, θ_min_ = 2.4°
ω scan	*h* = −11→11
Absorption correction: multi-scan (*CrysAlis PRO*; Agilent, 2011)	*k* = −13→18
*T*_min_ = 0.876, *T*_max_ = 0.956	*l* = −13→13
12068 measured reflections	

### Refinement

**Table:**

Refinement on *F*^2^	Primary atom site location: structure-invariant direct methods
Least-squares matrix: full	Secondary atom site location: difference Fourier map
*R*[*F*^2^ > 2σ(*F*^2^)] = 0.071	Hydrogen site location: inferred from neighbouring sites
*wR*(*F*^2^) = 0.215	H-atom parameters constrained
*S* = 1.06	*w* = 1/[σ^2^(*F*_o_^2^) + (0.1054*P*)^2^ + 2.1576*P*] where *P* = (*F*_o_^2^ + 2*F*_c_^2^)/3
3105 reflections	(Δ/σ)_max_ = 0.001
199 parameters	Δρ_max_ = 0.69 e Å^−^^3^
0 restraints	Δρ_min_ = −0.41 e Å^−^^3^

### Fractional atomic coordinates and isotropic or equivalent isotropic displacement parameters (Å^2^)

**Table:**

	*x*	*y*	*z*	*U*_iso_*/*U*_eq_	
S1	0.83675 (13)	0.46698 (8)	0.30886 (10)	0.0500 (3)	
S2	0.55748 (13)	0.61673 (8)	0.24923 (9)	0.0454 (3)	
F1	1.4449 (3)	0.9101 (2)	0.4037 (3)	0.0707 (8)	
F2	1.2781 (3)	1.0011 (2)	0.4699 (3)	0.0679 (8)	
F3	1.3609 (4)	1.0282 (2)	0.2933 (3)	0.0684 (8)	
O1	0.4182 (4)	0.6682 (2)	0.2516 (3)	0.0549 (8)	
O2	0.5788 (4)	0.5696 (2)	0.1331 (2)	0.0547 (8)	
O3	1.0875 (4)	0.8720 (2)	0.3708 (3)	0.0540 (8)	
O4	1.2090 (4)	0.8779 (3)	0.1895 (3)	0.0621 (9)	
N1	0.6932 (4)	0.4364 (2)	0.4965 (3)	0.0429 (8)	
N2	0.5655 (4)	0.5461 (2)	0.3653 (3)	0.0407 (8)	
H2	0.4899	0.5458	0.4127	0.049*	
C1	0.9139 (5)	0.3890 (3)	0.4238 (4)	0.0525 (11)	
H1	1.0078	0.3563	0.4215	0.063*	
C2	0.8241 (5)	0.3813 (3)	0.5146 (4)	0.0487 (10)	
H2A	0.8471	0.3419	0.5853	0.058*	
C3	0.6816 (5)	0.4886 (3)	0.3920 (3)	0.0393 (9)	
C4	0.7160 (5)	0.6916 (3)	0.2825 (3)	0.0390 (9)	
C5	0.8250 (5)	0.7001 (3)	0.1993 (3)	0.0444 (10)	
H5	0.8150	0.6645	0.1242	0.053*	
C6	0.9482 (5)	0.7601 (3)	0.2248 (3)	0.0422 (9)	
H6	1.0222	0.7663	0.1672	0.051*	
C7	0.9628 (4)	0.8114 (3)	0.3362 (3)	0.0356 (8)	
C8	0.8530 (5)	0.8018 (3)	0.4203 (3)	0.0408 (9)	
H8	0.8636	0.8362	0.4963	0.049*	
C9	0.7304 (5)	0.7431 (3)	0.3940 (3)	0.0414 (9)	
H9	0.6556	0.7374	0.4510	0.050*	
C10	1.1941 (5)	0.8985 (3)	0.2972 (4)	0.0484 (10)	
C11	1.3199 (6)	0.9613 (4)	0.3672 (5)	0.0544 (11)	

### Atomic displacement parameters (Å^2^)

**Table:**

	*U*^11^	*U*^22^	*U*^33^	*U*^12^	*U*^13^	*U*^23^
S1	0.0531 (7)	0.0573 (7)	0.0405 (6)	−0.0071 (5)	0.0083 (5)	−0.0031 (5)
S2	0.0539 (6)	0.0557 (6)	0.0244 (5)	−0.0088 (5)	−0.0045 (4)	0.0085 (4)
F1	0.0512 (16)	0.0725 (19)	0.086 (2)	0.0087 (14)	−0.0015 (14)	0.0160 (17)
F2	0.0624 (17)	0.081 (2)	0.0607 (17)	−0.0100 (15)	0.0071 (13)	−0.0193 (15)
F3	0.0685 (19)	0.0635 (18)	0.0730 (19)	−0.0053 (14)	0.0073 (15)	0.0189 (15)
O1	0.0508 (17)	0.073 (2)	0.0385 (15)	−0.0029 (15)	−0.0064 (13)	0.0167 (15)
O2	0.076 (2)	0.0631 (19)	0.0219 (13)	−0.0207 (17)	−0.0062 (13)	0.0018 (13)
O3	0.062 (2)	0.0564 (19)	0.0436 (16)	0.0032 (15)	0.0051 (14)	0.0015 (14)
O4	0.071 (2)	0.073 (2)	0.0442 (18)	−0.0039 (18)	0.0159 (15)	−0.0029 (16)
N1	0.059 (2)	0.0393 (18)	0.0288 (15)	0.0003 (16)	−0.0016 (14)	−0.0013 (13)
N2	0.0466 (18)	0.051 (2)	0.0247 (14)	−0.0051 (15)	0.0032 (13)	0.0077 (13)
C1	0.054 (3)	0.049 (2)	0.053 (3)	0.003 (2)	−0.002 (2)	−0.013 (2)
C2	0.063 (3)	0.042 (2)	0.038 (2)	0.003 (2)	−0.0043 (19)	−0.0034 (17)
C3	0.051 (2)	0.041 (2)	0.0248 (17)	−0.0051 (18)	−0.0011 (15)	−0.0027 (15)
C4	0.050 (2)	0.042 (2)	0.0229 (16)	−0.0013 (17)	−0.0038 (15)	0.0024 (15)
C5	0.063 (3)	0.048 (2)	0.0211 (16)	−0.004 (2)	0.0020 (16)	−0.0050 (15)
C6	0.054 (2)	0.047 (2)	0.0266 (17)	0.0037 (19)	0.0092 (16)	−0.0010 (16)
C7	0.046 (2)	0.0314 (18)	0.0282 (17)	0.0078 (16)	−0.0004 (15)	0.0033 (14)
C8	0.057 (2)	0.041 (2)	0.0239 (16)	0.0028 (18)	0.0035 (16)	−0.0042 (15)
C9	0.053 (2)	0.049 (2)	0.0231 (16)	−0.0024 (18)	0.0067 (15)	0.0023 (16)
C10	0.051 (2)	0.050 (2)	0.044 (2)	0.0062 (19)	0.0088 (19)	0.0039 (19)
C11	0.050 (3)	0.062 (3)	0.051 (3)	0.007 (2)	0.006 (2)	0.007 (2)

### Geometric parameters (Å, º)

**Table:**

S1—C3	1.738 (4)	N2—H2	0.8800
S1—C1	1.745 (5)	C1—C2	1.326 (6)
S2—O1	1.430 (4)	C1—H1	0.9500
S2—O2	1.450 (3)	C2—H2A	0.9500
S2—N2	1.604 (3)	C4—C5	1.386 (6)
S2—C4	1.762 (4)	C4—C9	1.400 (5)
F1—C11	1.339 (5)	C5—C6	1.385 (6)
F2—C11	1.331 (5)	C5—H5	0.9500
F3—C11	1.325 (5)	C6—C7	1.398 (5)
O3—C10	1.346 (5)	C6—H6	0.9500
O3—C7	1.414 (5)	C7—C8	1.399 (5)
O4—C10	1.214 (5)	C8—C9	1.370 (6)
N1—C3	1.344 (5)	C8—H8	0.9500
N1—C2	1.389 (6)	C9—H9	0.9500
N2—C3	1.317 (5)	C10—C11	1.550 (7)
			
C3—S1—C1	90.8 (2)	C6—C5—C4	120.4 (3)
O1—S2—O2	117.08 (19)	C6—C5—H5	119.8
O1—S2—N2	106.00 (19)	C4—C5—H5	119.8
O2—S2—N2	111.73 (19)	C5—C6—C7	119.4 (4)
O1—S2—C4	109.3 (2)	C5—C6—H6	120.3
O2—S2—C4	106.50 (19)	C7—C6—H6	120.3
N2—S2—C4	105.72 (17)	C6—C7—C8	119.8 (4)
C10—O3—C7	126.0 (3)	C6—C7—O3	122.8 (3)
C3—N1—C2	114.7 (4)	C8—C7—O3	117.3 (3)
C3—N2—S2	122.1 (3)	C9—C8—C7	120.6 (3)
C3—N2—H2	119.0	C9—C8—H8	119.7
S2—N2—H2	119.0	C7—C8—H8	119.7
C2—C1—S1	111.0 (4)	C8—C9—C4	119.5 (4)
C2—C1—H1	124.5	C8—C9—H9	120.2
S1—C1—H1	124.5	C4—C9—H9	120.2
C1—C2—N1	113.6 (4)	O4—C10—O3	130.4 (5)
C1—C2—H2A	123.2	O4—C10—C11	117.0 (4)
N1—C2—H2A	123.2	O3—C10—C11	112.6 (4)
N2—C3—N1	121.0 (4)	F3—C11—F2	107.4 (4)
N2—C3—S1	129.1 (3)	F3—C11—F1	107.9 (4)
N1—C3—S1	109.9 (3)	F2—C11—F1	106.9 (4)
C5—C4—C9	120.2 (4)	F3—C11—C10	111.3 (4)
C5—C4—S2	120.6 (3)	F2—C11—C10	113.9 (4)
C9—C4—S2	119.2 (3)	F1—C11—C10	109.2 (4)
			
O1—S2—N2—C3	−178.9 (3)	S2—C4—C5—C6	179.0 (3)
O2—S2—N2—C3	52.5 (4)	C4—C5—C6—C7	0.7 (6)
C4—S2—N2—C3	−63.0 (4)	C5—C6—C7—C8	−0.1 (6)
C3—S1—C1—C2	0.9 (4)	C5—C6—C7—O3	178.1 (3)
S1—C1—C2—N1	−0.1 (5)	C10—O3—C7—C6	9.8 (6)
C3—N1—C2—C1	−1.1 (5)	C10—O3—C7—C8	−171.9 (4)
S2—N2—C3—N1	173.6 (3)	C6—C7—C8—C9	−0.6 (6)
S2—N2—C3—S1	−6.0 (5)	O3—C7—C8—C9	−178.9 (3)
C2—N1—C3—N2	−178.0 (4)	C7—C8—C9—C4	0.7 (6)
C2—N1—C3—S1	1.7 (4)	C5—C4—C9—C8	−0.1 (6)
C1—S1—C3—N2	178.2 (4)	S2—C4—C9—C8	−179.7 (3)
C1—S1—C3—N1	−1.5 (3)	C7—O3—C10—O4	−0.2 (8)
O1—S2—C4—C5	−123.6 (3)	C7—O3—C10—C11	−176.8 (3)
O2—S2—C4—C5	3.7 (4)	O4—C10—C11—F3	41.5 (6)
N2—S2—C4—C5	122.7 (3)	O3—C10—C11—F3	−141.4 (4)
O1—S2—C4—C9	56.0 (4)	O4—C10—C11—F2	163.1 (4)
O2—S2—C4—C9	−176.7 (3)	O3—C10—C11—F2	−19.8 (5)
N2—S2—C4—C9	−57.7 (4)	O4—C10—C11—F1	−77.6 (5)
C9—C4—C5—C6	−0.6 (6)	O3—C10—C11—F1	99.5 (4)

### Hydrogen-bond geometry (Å, º)

**Table:**

*D*—H···*A*	*D*—H	H···*A*	*D*···*A*	*D*—H···*A*
N2—H2···N1^i^	0.88	1.99	2.858 (5)	171

Symmetry code: (i) −*x*+1, −*y*+1, −*z*+1.
